# Up-to-date on mortality in COPD - report from the OLIN COPD study

**DOI:** 10.1186/1471-2466-12-1

**Published:** 2012-01-09

**Authors:** Anne Lindberg, Lars-Gunnar Larsson, Hana Muellerova, Eva Rönmark, Bo Lundbäck

**Affiliations:** 1 Department of Public Health and Clinical Medicine, Division of Medicine, Umeå University, SE-901 85 Umeå, Sweden; 2 The OLIN studies and Division of Respiratory Medicine & Allergy, Sunderby Hospital, SE-971 80 Luleå, Sweden; 3 Division of Respiratory Medicine & Allergy, Sunderby Hospital, SE-971 80 Luleå, Sweden; 4 WorldWide Epidemiology, GlaxoSmithKline R&D, Stockley Park, Uxbridge, Middlesex, UB11 1BT, UK; 5 Department of Public Health and Clinical Medicine, Division of Occupational and Environmental Medicine, Umeå University, Umeå, SE-901 85 Sweden; 6 Department of Internal Medicine/Krefting Research Centre, Sahlgrenska Academy, University of Gothenburg, SE-405 30 Gothenburg, Sweden

## Abstract

**Background:**

The poor recognition and related underdiagnosis of COPD contributes to an underestimation of mortality in subjects with COPD. Data derived from population studies can advance our understanding of the true burden of COPD. The objective of this report was to evaluate the impact of COPD on mortality and its predictors in a cohort of subjects with and without COPD recruited during the twenty first century.

**Methods:**

All subjects with COPD (n = 993) defined according to the GOLD spirometric criteria, FEV_1_/FVC < 0.70, and gender- and age-matched subjects without airway obstruction, non-COPD (n = 993), were identified in a clinical follow-up survey of the Obstructive Lung Disease in Northern Sweden (OLIN) Studies cohorts in 2002-2004. Mortality was observed until the end of year 2007. Baseline data from examination at recruitment were used in the risk factor analyses; age, smoking status, lung function (FEV_1 _% predicted) and reported heart disease.

**Results:**

The mortality was significantly higher among subjects with COPD, 10.9%, compared to subjects without COPD, 5.8% (p < 0.001). Mortality was associated with higher age, being a current smoker, male gender, and COPD. Replacing COPD with FEV_1 _% predicted in the multivariate model resulted in the decreasing level of FEV_1 _being a significant risk factor for death, while heart disease was not a significant risk factor for death in any of the models.

**Conclusions:**

In this cohort COPD and decreased FEV_1 _were significant risk factors for death when adjusted for age, gender, smoking habits and reported heart disease.

## Background

Chronic Obstructive Pulmonary Disease (COPD) is recognized as a major public health problem with an increasing morbidity and mortality. It has been forecasted that COPD will be ranked the fourth burden of disease worldwide by year 2030 [1]. The prevalence of COPD is most often reported in the range of 6-10% of the total adult population [2], it is strongly correlated to smoking and age, and about 50% of elderly smokers fulfil the spirometric criteria of COPD [3]. Population studies have shown that a large majority of COPD patients have mild-to-moderate disease [4].

The underdiagnosis of COPD is well-known. Only about a third of all cases with COPD have been recognized by the health care [3-6], and the proportion of undiagnosed cases decreases with increasing disease severity [4]. Most reports on mortality in COPD are based on death certificates and hence, due to the underdiagnosis, the true impact of COPD on mortality is probably greatly underestimated. There are only few reports on mortality in COPD based on population studies. In a follow-up over up to 22 years of a large general population cohort in the USA, the NHANES I recruited 1971-75 follow-up in 1992 included totally 923 cases of COPD. The overall mortality in COPD was 44% and in severe COPD 71%. Subjects with mild, moderate and severe COPD, and subjects with restrictive lung function impairment, had an increased risk for death [7]. There is a 30-year follow up of a large population sample from southern Sweden, both smokers and non-smokers with COPD had a significantly increased risk for death [8]. The first cohort of the Obstructive Lung Disease in Northern Sweden (OLIN) studies, was recruited in 1985-86, and in a recently published 20-year follow-up an overall mortality of 54% in subjects with COPD was reported, while the mortality in severe and very severe COPD at entry was 81% [9].

Irrespective of COPD, decline in lung function, expressed as FEV_1_, is known to predict death [10,11]. Reduced FEV_1 _has also been reported to be a marker of cardiovascular death [12]. However, due to the underdiagnosis and lack of longitudinal epidemiological data, the present impact of COPD identified through spirometric screening in the general population on mortality considering the influence of other factors such as cardiovascular co-morbidity has been ill-described.

Within the OLIN studies, cross-sectional and longitudinal data on respiratory diseases, including lung function, have been collected in several cohorts recruited from the general population at different occasions ever since 1985 [3,4,9,13,14]. Previously recruited adult OLIN-cohorts were invited to a clinical follow-up in 2002-2004, and from the participants all subjects with COPD were identified together with age and gender matched subjects without airway obstruction [15]. The aim of the present paper is to report overall mortality in this cohort based on mortality data collected up to the end of 2007, and to evaluate the impact of COPD, level of FEV_1_, gender, smoking habits and heart disease on mortality.

## Methods

### Study design and Study population

The recruitment of the study cohort and the study design has been reported previously [15]. In 2002-2004 four previously identified population based adult OLIN cohort (one from the eighties and three from the nineties) were invited to re-examination including structured interview and spirometry. When the examinations were completed all subjects with COPD according to The Global Initiative for Chronic Obstructive Lung Disease (GOLD) spirometric criteria [16], FEV_1_/FVC < 0.70, were identified (n = 993) together with an age- and gender matched reference sample with non-obstructive lung function. This cohort, all together 1986 subjects (1084 men; 902 women) has since 2005 been invited to yearly examinations including lung function testing and a structured interview. The study was approved by the Regional Ethics Committee at University Hospital of Northern Sweden and Umeå University.

Data collected in 2002-2004 were used for baseline characteristics. The observation time is from the date of examination at recruitment until the end of year 2007, and the mean follow-up time can be approximated to four years. Mortality data during the period, including date of death, were collected from the national mortality register.

### Baseline measurements

#### Structured interview

A previously validated questionnaire was administered via a structured interview [4,13,14]. Smoking habits were classified into the following groups: non-smokers, ex-smokers (stopped at least one year before the baseline visit) and current smokers. The variable 'heart disease' includes self-reported angina pectoris, previous coronary artery bypass surgery, previous percutaneous coronary intervention, myocardial infarction or heart failure.

#### Spirometry

The lung function tests were performed using a dry spirometer, Mijnhardt Vicatest 5 by following the ATS guidelines [17]. Vital capacity (VC) was defined as the best value of forced vital capacity (FVC) and slow vital capacity (SVC). A reversibility test was performed when the ratio of FEV_1_/VC was < 70% or if FEV_1 _was < 90% of predicted value. The Swedish normal values by Berglund et al were used [18]. When calculating the FEV_1_/VC ratio and defining the severity of COPD, the largest value of FEV_1 _as well as of VC was used.

#### Body Mass Index

Weight and height were recorded before the lung function test. Body Mass Index (BMI) was calculated: weight (kg)/(height (m)*height (m)) and was classified into normal (range 20 - < 25), underweight (< 20), overweight (range 25 - < 30) and obesity (≥ 30).

#### Classification of COPD

Spirometric classification of COPD according to GOLD, FEV_1_/VC < 0.7, was used. The classification of severity of COPD includes for stages based on FEV_1 _% predicted.

Stage I FEV_1 _≥ 80% predicted

Stage II FEV_1 _≥50 and < 80% predicted

Stage III FEV_1 _≥30 and < 50% predicted

Stage IV FEV_1 _< 30% predicted

### Statistical analysis

Statistical calculations were made using the Statistical Package for the Social Sciences (SPSS) software version PASW 18.0 and Microsoft Excel 2007. The sample comprised of six age groups. Crude mortality rates based on data collected by the end of 2007 were analysed by gender, disease status (COPD or non-COPD), and selected baseline descriptors (smoking habits, BMI and heart disease). The chi-squared test was used for bi-variate comparisons and to test for trends. A p-value of < 0.05 was regarded as significant. The hazard function of death, which describes the momentary risk, was estimated by use of Poisson regression depending on several variables. The follow-up period of each individual was divided into intervals of the length 0.1 years, when the contribution to the log likelihood function was calculated. The variables age and observation time was updated at each interval. For each risk variable of death, we calculated the hazard ratio (HR) of an increase of 1 unit and the corresponding 95% confidence interval (CI). Three different models were studied including the following covariates: [1] age, observation time, gender, heart disease and disease status (COPD or non-COPD) [2] age, observation time, gender, disease status (COPD or non-COPD), heart disease, and FEV_1 _percent predicted, [3] age, observation time, gender, heart disease, and FEV_1 _percent predicted. For the models [1] and [3] analyses were also done including the variable smoking habits categorized as non-smoker, ex-smoker and smoker. A possible interaction between COPD and heart disease was tested in model 1 and an interaction between heart disease and FEV_1 _in the later models. Multivariable analyses were also conducted in models including the co-variates BMI and smoking categories. Furthermore, we used a Poisson regression model including spline functions of FEV_1_, which allowed the hazard function to vary with a greater freedom and still correspond to a smooth curve. A simple model includes just one coefficient of FEV_1 _and that restrict the shape of the curve.

## Results

### Study population

Baseline characteristics of the study population at recruitment are shown in table 1. All together, 36.2% of the subjects were non-smokers, 40.5% ex-smokers and 23.3% current smokers. There were more smokers and fewer non-smokers in subjects with COPD compared to in non-COPD. The prevalence of self-reported heart disease was similar in COPD and non-COPD (p = 0.429). At recruitment the distribution by disease severity among subjects with COPD was 52.2%, 40.3%, 6.1%, and 1.4%, in GOLD stages I, II, III, and IV respectively.

**Table 1:** Study population, basic characteristics at the recruitment (2002-2004)

Category	Variable	COPD (n = 993)	No COPD (n = 993)
Gender (n)	Men	543	541
	Women	450	452
Age group, year of birth (n)	-1920	113	113
	1921-30	146	147
	1931-40	374	358
	1941-50	267	282
	1951-60	73	73
	1961-	20	20
Smoking (%)	Non smoker	24.1	47.9
	Ex smoker	41.5	39.6
	Smoker	34.4	12.6
BMI (%)	< 20	3.9	1.6
	20 - < 25	40.8	35.6
	25 - < 30	39.9	46.5
	≥ 30	15.3	16.4
Heart disease (%)	Yes	16.9	14.6

### Mortality

In total, 166 subjects, 8.4% (9.6% men; 6.9% women, p = 0.029), had died until the end of 2007. Crude mortality data are shown in table 2. The mortality was significantly higher among subjects with COPD, 10.9% (n = 108), compared to non-COPD subjects, 5.8% (n = 58) (p < 0.001). No one had died in the youngest age group (born after 1961) but thereafter the mortality increased by increasing age both among subjects with and without COPD. Comparing COPD and non-COPD, the mortality was significantly higher among subjects with COPD in the three oldest age groups (born 1920 and before, 1921-1930 and 1931-1940). In smokers and ex-smokers, in subjects with BMI > 25, and among those without concomitant heart disease, the mortality was significantly higher in subjects with COPD as compared to non-COPD. There was no significant difference in mortality between the COPD and non-COPD groups among non-smokers, subjects with underweight or normal weight (BMI < 20 or BMI 20-25), and among subjects with reported heart disease.

**Table 2:** Crude mortality in percent among subjects with COPD and controls (FEV_1_/VC < 0.70 and FEV_1_/VC > 0.70, respectively), by gender, age-group (year of birth), smoking habits, BMI and reported heart disease

Category	Variable	**COPD **(n = 993)	p-value^1^	**No COPD **(n = 993)	p-value^2^	p-value^3^
Gender	Men	12.3		6.9		0.002
	Women	9.1	0.109	4.6	0.138	0.008
Age group, born	-1920	32.7		15.9		0.003
	1921-30	18.4		9.6		0.030
	1931-40	9.1		4.7		0.020
	1941-50	3.0		3.2		0.883
	1951-60	2.8		0		
	1961-	0		0		
Smoking	Non smoker	8.3		5.3		0.125
	Ex smoker	12.2		6.7		0.008
	Smoker	11.3	0.314	4.8	0.617	0.036
BMI	< 20	25.6		25.0		0.960
	20 - < 25	9.9		6.8		0.131
	25 - < 30	9.6		4.8		0.006
	≥ 30	12.5	0.016	4.3	0.004	0.009
Heart disease	No	9.1		4.5		< 0.001
	Yes	19.6	< 0.001	12.9	< 0.001	0.102

^1^Comparing within each category among subjects with COPD

^2^Comparing within each category among controls (no COPD)

^3^Comparing COPD and controls (no COPD) by each variable

#### Analyses of risk factors for death

**Figure 1:**
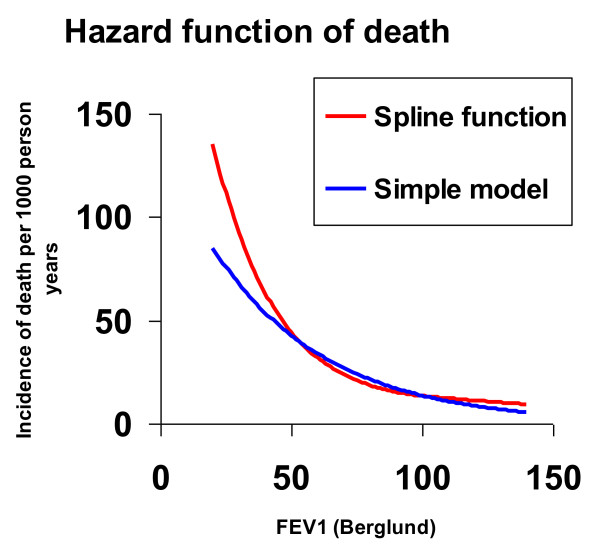
**Hazard function of death, expressed as incidence of death per 1000 person years, by FEV_1 _percent predicted in a 70 year old man after two years observation time in a model using a spline function and a simple model**.

In a multivariate model, COPD was a significant risk factor for death (HR 2.06, CI 1.49-2.85), and so was increasing age (HR 1.08, CI 1.07-1.10), male gender (HR 1.52, CI 1.10-2.10) and heart disease (HR 1.43, CI 1.01-2.02). When the variable smoking habits was added to the model, heart disease did not reach statistical significance, but close to (HR 1.42, CI 1.00-2.01) (table 3). BMI did not contribute significantly and ex-smoker did not significantly differ for non-smoker (not shown in tables). No significant interaction was found between heart disease and COPD or between FEV_1 _and heart disease, thus COPD was a significant risk factor for death irrespectively of reported heart disease at start of the observation period. Nor was there any significant interaction between COPD and smoking, i.e. COPD was associated with an increased mortality independent if smoking or not. In a model including both COPD and FEV_1_, COPD lost its significance as a risk factor for death in the presence of FEV_1 _in the statistical model. Figure 1 illustrates an example of the hazard function of death in relation to FEV_1 _in a 70 year old man after an observation time of two years. The model including a spline function indicates that the effect of FEV_1 _on mortality is more outstanding in subjects with FEV_1 _< 50% of predicted values than what can be shown by using a simpler model. Using FEV_1 _instead of COPD in the multivariate model, decrease in level of FEV_1 _at baseline was an independent significant risk factor for death together with increasing age, male gender and current smoking while heart disease did not reach statistical significance (table 4).

**Table 3:** Risk factors for death expressed as Hazard Ratio (HR) and 95% Confidence Interval (CI) including the co-variates age, time since recruitment, gender, smoking habits, COPD and heart disease.

Variable	HR	95% CI	p-value
Age^1^	1.09	1.07-1.11	< 0.001
Time since recruitment^1^	1.03	0.95-1.12	0.504
Gender^2^	1.43	1.02-2.00	0.040
Ex-smoker^3^	1.29	0.87-1.92	0.203
Smoker^3^	2.01	1.27-3.19	0.003
Heart disease^4^	1.42	1.00-2.01	0.051
COPD^4^	1.77	1.26-2.49	0.001

^1^One unit change represents one year

^2^Reference group: female gender

^3^Reference group: non smoker

^4^Reference group: not reporting heart-disease respectively non-COPD

**Table 4:** Risk factors for death expressed as Hazard Ratio (HR) and 95% Confidence Interval (CI), including the co-variates age, time since recruitment, gender, heart disease, smoking habits and FEV_1 _percent predicted.

Variable	HR	95% CI	p-value
Age^1^	1.09	1.07-1.11	< 0.001
Time since recruitment^1^	1.03	0.95-1.12	0.478
Gender^2^	1.45	1.03-2.03	0.034
Ex-smoker^3^	1.26	0.85-1.86	0.259
Smoker^3^	1.91	1.21-2.99	0.005
Heart disease^4^	1.36	0.96-1.93	0.084
FEV_1 _percent predicted^5^	0.98	0.97-0.99	< 0.001

^1^One unit change represents one year

^2^Reference group: female gender

^3^Reference group: non-smoker

^4^Reference group: not reporting heart-disease

^5^The unit change represent one percent unit

## Discussion

The strength and newsworthy of the current study is the up-to date report on the impact of COPD on mortality. The identification of the study population and the observation time took place during the twenty first century when treatment for COPD as well as cardiovascular disease was recommended according to modern and current guidelines. Further, the distribution of disease severity among subjects with COPD in the cohort was representative for what has been reported for the general population, comprising of more than 90% of patients in GOLD stages I and II [4]. COPD was a significant risk factor of increased mortality in this topical epidemiological context.

There are only a few previously published population based studies on mortality in subjects with COPD defined according to currently accepted spirometric criteria [7-9,19]. As referred to in the introduction, these study populations were recruited during the nineteen-seventies and eighties. The mortality data in these studies were based on long term follow-ups from about ten years to more than twenty years, thus a healthy survivor effect must be taken into account when evaluating these data. Further, updated guidelines during the last decade for treatment of not only COPD, but also cardiovascular disease, have improved the prognosis compared to thirty years ago. Consequently, the starting point, the time and the length of the observation time are of importance when evaluating and comparing mortality data not only in COPD but also other diseases. There are mortality data collected in two large clinical trials started during the nineties with an observation time over three and four years respectively, the TORCH and the UPLIFT studies. The all-cause mortality reported from the TORCH-study was 14.3% [20] in a population with moderate- to severe COPD (FEV_1 _< 60 percent of predicted). In the UPLIFT-study [21] subjects with a post-bronchodilator FEV_1 _< 70 percent of predicted were included, and the crude mortality in the total population was 15.4% at the end of the treatment period. After the approximately four year's observation time, similar to the UPLIFT, the mortality was 10.9% among subjects with in COPD in our study. However, the UPLIFT-cohort had a lower baseline mean FEV_1 _compared with our study, further, as most clinical studies the UPLIFT-study included a selected population with regard not only to lung function but also to other factors such as age and co-morbidity. Further, in a 13-year follow-up of the ISOLDE study [22], the mortality was 56% in the study population including subjects with moderate- to severe COPD.

The reported mortality in this study can be considered up-to-date as both the identification of the study population and the observation time took place after the turn of the century, where modern and current guidelines for treatment have been well established in Sweden. Subjects with COPD in the cohort are considered representative for the general population with regard to distribution by disease severity [4]. There are, to the best of our knowledge, no other published similar studies. Further strengths of the study are the large size of the COPD-population, comparable to that of the NHANES I [7], the accuracy of mortality data and that there was no loss of follow-up. However, possible limitations are the comparatively short time of follow up and that information on heart disease was self-reported and not collected from medical records. Another limitation is that the classification of COPD was strictly made by spirometric criteria without regard to respiratory symptoms. This has to be considered when interpreting the data, as respiratory symptoms are known to affect the prognosis in mild/moderate COPD [23]. Further, the non-COPD population did also include subjects with restrictive lung function impairment, even though the ability for a dynamic spirometry to identify restrictive lung function can be questioned. The reasons for a restrictive pattern on dynamic spirometry are highly heterogeneous and reflect different underlying disorders as idiopathic pulmonary fibrosis, thoracic deformities, obesity, pleural effusion, cardiac insufficiency and neuromuscular diseases.

As expected, increasing age was the most prominent risk factor for death. Among all subjects born < 1940, the proportion of deceased was significantly larger among subjects with COPD. The use of a fixed ratio to define airway obstruction, FEV_1_/FVC < 0.70, has been discussed with regard to identifying clinically relevant COPD among elderly [24], but the results from our study indicate that the fixed ratio identifies a population with significantly increased mortality also among subjects older than 80 years. There is a recent report on 5-year mortality among subjects aged > 65 years, where COPD according to GOLD was not associated with increased mortality among those older then 75 years [25]. However, the study population was recruited was from an out patient clinic, and can thus not be considered to reflect COPD in the general population.

When FEV_1 _was included in the multivariate analyse model, COPD was no longer a significant risk factor for death, but the level of FEV_1_, reflecting disease severity at recruitment, was related to mortality. Our data exemplify that a decrease in FEV_1 _in the range of 100 to 50 percent of predicted will continuously increase the risk for death, and further, illustrated by Figure 1, a dramatic increased risk for death occurs when FEV_1 _continues to decrease below 50 percent of predicted. It is well-known that tobacco exposure contributes to the development of both COPD and cardiovascular diseases, and cardiovascular death is the most common cause of death in the world. In a multivariate model heart disease was a significant risk factor for death just as COPD, age and male gender, however, when smoking habits were added to the model there was a slight change. Smoking roughly doubled the risk for death while male gender and reported heart disease each increased the risk on a similar level, approximately 40%, even though heart disease did not reach statistical significance as a risk factor. Impaired lung function is a known risk factor for death [10,11] and according to our results the risk for death in subjects with COPD, when adjusted for confounders including presence of heart disease, was increased by about 75% compared to in subjects without COPD. Maybe the impact of current treatment guidelines of cardiovascular disease has reduced mortality contributing to the borderline significance of heart disease as a risk factor while we found COPD and impaired lung function still being strong and significant risk factors for death.

Besides smoking, BMI is a known prognostic factor in COPD, and increased loss of weight is associated with a higher mortality in COPD [26,27]. In this study BMI could not predict mortality; however, longitudinal data on weight loss were not included. An important message is also the benefit of smoking cessation, i.e. being an ex-smoker did not differ significantly from non-smokers with regard to risk for death, while current smoking roughly doubled the risk for death. According to a 9-year follow-up from the ECRHS non-smoking non-symptomatic young adults with mild/moderate COPD do not have worse outcome than subjects without COPD [23]. Further follow-up of our cohort will give us corresponding data from middle aged and elderly subjects with mild/moderate COPD. There was surprisingly no significant difference in prevalence of heart disease in subjects with and without COPD, however, the dominance of GOLD stage I and II in the COPD-cohort might contribute to this finding.

## Conclusions

In this recently identified cohort, where COPD was mostly represented by GOLD stages I & II, COPD, age and current smoking were the strongest risk factors for death. Male gender and reported heart disease were also, and on a similar level, associated to an increased risk for death, even though heart disease did not reach statistical significance. The results further indicate that not only COPD but also impaired lung function, expressed as level of FEV_1_, is a significant risk factor for death independent of confounders as age, gender, smoking habits and heart disease.

## Competing interests

The authors declare that they have no competing interests. Hana Muellerova is an employee of GlaxoSmithKline, R&D, a producer of pharmaceuticals and owns shares and stock options of GlaxoSmithKline plc. The authors alone are responsible for the content and writing of the paper.

## Authors' contributions

AL designed the study, performed the statistical analyses, drafted and revised the manuscript. LGL and HM contributed to the paper by interpretation of data and critically revision of the manuscript. ER participated in the design of the study, and contributed to the paper by interpretation of data and critically revision of the manuscript. BL designed the study, drafted the manuscript, contributed to the paper by interpretation of data and critically revision of the manuscript.

All authors have read and approved the final manuscript.

## Pre-publication history

The pre-publication history for this paper can be accessed here:

http://www.biomedcentral.com/1471-2466/12/1/prepub
